# On Some Points Having Reference to the Differential Diagnosis of Fibrous Tumors of the Uterus

**Published:** 1865-04

**Authors:** C. H. F. Routh

**Affiliations:** Physician to the Samaritan Hospital for Women and Children


### ON SOME POINTS HAVING REFERENCE TO THE DIFFERENTIAL DIAGNOSIS OF FIBROUS TUMORS OF THE UTERUS.

By Dr. C. H. F. ROUTH, Physician to the Samaritan Hospital for Women and Children.

The symptoms of fibrous tumor most dwelt upon in speaking of the disease are an enlarged abdomen, with a hard, rounded and solid tumor to be felt through the abdominal parietes in and about the uterine region, extending sometimes as high as the epigastric region, both lumbar regions being clear on percussion, and the umbilicus sunken in, not projecting. The tumor is also to be felt *per vaginam*, involving the uterus; an occasional loud and systolic souffle is to be heard over the tumor; menorrhagia is more or less intense; there is absence of fluctuation; the length of the uterine cavity is increased; and, lastly, owing to the close union of the uterus and bladder, the growth is drawn so near the abdominal walls, that it precludes the passage of the hand low down between the tumor and the walls of the abdomen. I will speak of these symptoms as I go on, to test their relative value.

1. *Physical Hardness, Roundness, and Solidity of the Tumor.*—The feel of a fibrous tumor has been considered by many as almost pathognomonic. This, in connection with its rounded and lobulated character, is such, that the combination of the two characteristics would seem to make error almost an impossibility. Any one, however, accustomed to examine the cases of cellulitis and hæmatocele, must know the contrary. I know that the same thing has been observed in cases of ovarian dropsy, and it may be laid down as a general proposition, that a *thin* fluid, and *a fortiori* a *viscid* one, may be included in an inelastic bag and convey to the touch the impression of a solid.

I will illustrate this law by an experiment; and then mention a remarkable example in the living subject. Here is an ox's bladder contained within a calico bag. This bladder has been kept in spirit for a week, and is quite inelastic. The calico bag I put around it lest the bladder should burst. This bladder is now full of water, and fluctuation is quite evident everywhere. At its opening, however, you see I have affixed a tube connected with this pump, by which I can force in water *ad libitum*, securing its retention by this stopcock in the connecting tube. As I pump in water, you will find that fluctuation will gradually cease, till none of you shall be able to detect it; on the contrary, it will feel quite like a solid. If this be so with so thin a covering as this membrane and piece of calico, *a fortiori* will it be difficult in many cases to recognize fluctuation through the abdominal parietes; for I am quite sure fluids are contained within cavities of the body quite as tensely as in this bladder.

The following case, however, is so satisfactory on this point, that I cannot but mention it:—

An inmate of Bedlam Hospital, about thirty-five, declared herself pregnant; and Mr. Lawrence requested Dr. Greenhalgh to see her. Dr. Greenhalgh kindly allowed me to accompany him in his visit. We found a woman with a large tumor extending to above the umbilicus, very hard, nodulated, and giving altogether the impression of a solid tumor. The breasts, however, gave no indication of pregnancy. An examination *per vaginam* conveyed the same impression. The os, however, was hard; evidently that of an unimpregnated woman, and high up. The enlargement was chiefly in front of the uterus; and conveyed generally the same fibrous sensation. She passed urine freely; and, indeed, some was shown to us. On more carefully examining the tumor *per vaginam*, we thought that at one part we could detect some very obscure fluctuation, or at least a part less hard than others. It was thought wise that the bladder should be emptied of its contents. This was done; and about a chamber-pot and a-half of thickish urine was drawn. On examining the abdomen after the operation, the tumor was gone; and yet, had we trusted to mere manipulation, both Dr. Greenhalgh and I would almost have positively asserted that, if ever we had before felt a fibrous tumor, this was one.

2. *The Auscultatory Signs of an Uterine Fibrous Tumor* are four in number:—*a.* Two souffles, one a tubular, another a vesicular murmur; *b.* A thrill; *c.* A single or double cardiac sound; *d.* Absence of multilocular arrangement indicated on percussion.

*a.* Fibrous tumors ars sometimes the seat of a souffle. Most writers do not discriminate between the two, and appear to refer them to the same cause. Thus it is described by Dr. M'Clintock as always

"Synchronous with the pulse; sometimes short and abrupt, a mere whiff accompanying each arterial pulsation; at other times, prolonged and musical, and not to be distinguished by the most acute and practical ear from the *bruit placentaire.* Like it, it is occasionally loud and intense for some pulsations; then becoming feeble and almost inaudible. Again, it can be diminished and suppressed by moderate pressure of the stethoscope over the spot. It is present in those who have sustained no loss of blood, as well as in the anæmic." He adds that, "although a very interesting phenomenon, it is not one of any special diagnostic value, being common to pregnancy and ovarian disease, which are the two conditions most likely to be mistaken for fibrous tumor of the uterus." (M'Clintock's *Diseases of Women*, p. 131.)

However unwilling to differ from so high an authority, I cannot but take exception to the sweeping conclusion drawn. Nothing can be more graphic and true than the greater part of this description; but I think the fact is, that two very different souffles are here spoken of. The true *tubular* souffles are, so far as I know, very rarely, if ever heard in pregnancy and ovarian disease. The *bruit placentaire*, or one very like it, may be, perhaps often is.

Now, I believe that these two souffles are produced by two different causes. The first, or *vesicular*, *bruit placentaire*, if you like, is produced by the combined action of the large vessels immediately coming into and supplying the organ. The latest writer on the uterine appendages, Dr. Savage, believes these to be the only cause of souffle in fibroids, which are supplied largely by veins and and arteries, usually at each side.

But, I imagine, these produce only the vesicle murmur. The same cause produces them in pregnant uteri; and it is not due to placenta, since it is heard after delivery, as long as the vessels supplying it are in the hypertrophied condition. It was long since shown by Nægele (*On Obstetric Auscultation*, 1838), that in aneurismal varix, where a direct communication for the blood exists, so that it passes from the artery to the vein, the opposed or interfering currents, gave rise to a sound which may exactly resemble the *bruit placentaire.* The sound is also heard loudest at the point where the uterine arteries, reaching the broad ligament, become thicker in diameter, more tortuous, and sink into the uterus. How far the connexion between the arteries and veins in the pregnant or hypertrophied uterus may assist in the production of this sound, or whether the general circulation throughout the uterus must be taken into account, because so extensive, is a point for future inquiry. If we consider, however, that the arteries in the hypertrophied organ directly communicate with the large uterine veins; either, according to the Hunters, through the cavernous structure of the placenta; or, according to Webber, through the network of collossal capillaries; or, according to Goodsir, through a great cavity, which is everywhere traversed or intersected by filamentous prolongations of the uterine veins—we see how, physically, a sound like that of aneurismal varix could be produced by causes somewhat analogous. The later researches of Dr. Savage will rather explain the production of the sound from an extended circulation, if we assume the same structure to be present in the impregnated organ, only more extended in the hypertrophied or pregnant uterus. Now, it is unquestionable that, in many cases of fibrous tumors at their connection with the uterus, we have, as in pregnant uteri, elephantine venous sinusis. Such was found to be the case at the *post mortem* examination of Dr. Matthews Duncan's example of fibrous tumor, to be hereafter noticed.

This uterine murmur, or *bruit placentaire*, in either case originating from the same cause, is destroyed by pressure, if this be sufficiently strong; it may be by the stethoscope; it may be by intense uterine contraction, particularly in the vicinity and all around the *locale* of its production, exactly as it is abolished at the height of an uterine pain. Auscultation proves, then, that the souffle, after gradually rising in pitch up to the moment of greatest uterine contraction, becomes at last inaudible, except at the groins, where it is still heard, though more feebly so. For the same reasons, also, it is not heard immediately after delivery, when the uterus is firmly contracted, but again becomes audible in twenty minutes or half an hour afterwards, when, the uterine muscles having somewhat relaxed, the vessels again enlarge; a state which persists, except at intervals, for 48 hours or so, till such time as, in the process of involution, the vessels become permanently smaller. This, then, is one souffle which is occasionally heard in fibroids, and in every way resembles the placental souffle; and it is heard loudest at the groins, or over the entrance of the uterine arteries.

The *tubular* souffle, however, is different. It bears the same relation to the former as bronchial respiration does to vesicular, and is, I believe, due to pressure upon the aorta, and direct transmission of the aortal sounds through the fibrous growth. It needs only the continuity of a solid body between the aorta and abdominal parietes, and the aorta to be slightly compressed thereby, to give rise to these sounds.

A remarkable case, illustrative of this explanation, is given by M. Hérard (*Bull. Soc. Anat.* xxv, p. 148), where a very thin woman complained much of epigastric pulsations. There was no tumor; but ascultation revealed a souffle and a thrill. The case was diagnosed as one of aneurism. She died shortly of phthisis. The *post mortem* examination revealed a voluminous and also indurated left lobe of the liver. This it was that transmitted the aortic beat, and, by expression, produced the souffle.

We have, doubtless, all met with analogous cases, particularly in weak females, and especially where the middle lobe of the liver was indurated. Pressure over any large artery by a stethoscope will give rise to the same sound.

The position in which this sound is heard loudest also varies from that where the vesicular murmur is most audible. This is strongest at the inguinal region; the tubular souffle is generally heard loudest at some prominent point of the tumor, or within the vagina upon the inferior portions of the growth. It requires also some strength to compress the aorta through a large fibrous tumor; and hence one reason why it is rarely, if ever, destroyed by pressure of the stethoscope.

*b.* This tubular souffle is occasionally accompanied by a *thrill.* This thrill is very similar to that which is communicated to the ear in the case of an aneurism of the aorta, as M. Hérard's case proves. Indeed, in well marked cases, the musical note which accompanies the souffle and thrill are so exactly alike the sounds heard in cases of aneurism in the neighborhood of the heart, that it would seem impossible to detect a difference. Now, so far as I am aware, a thrill is never felt either in ovarian disease or in pregnancy. This is also the case in regard to the musical note. Once only have I heard this sound in the case of pregnancy. Then it was heard with the stethoscope upon the sacral region, the patient lying on her abdomen; and we can understand this. The aorta was pressed upon by the enlarged uterus, and the musical note, which could not be transmitted through the liquor amnii, was not heard in front. Behind, through the solid sacrum, it was readily heard. So far, this exceptional instance is a good explanation of its occurrence in fibroids.

*c.* Closely related to the tubular souffle is the *single* and *double* cardiac sound. This one sign, revealed by auscultation, I think most important; the more so, as it has not been hitherto sufficiently dwelt upon.

Let us remember that a fibrous tumor is, in most cases, a solid tumor. If it be of moderate size, and in any way can be or is compressed against the aorta directly or through the uterus—if the fibroid be placed in front of the organ—then the sounds of the aorta should be transmitted through the tumor. I do not know why it is; but these sounds are observed in practice to be of two kinds; first, a *single* sound synchronous with the systole of the heart; secondly, two sounds synchronous with and representing the double sound of the organ. I believe that whenever either of these sounds occur, you have a solid tumor between your stethoscope and the aorta; and so that it is a favorable argument, *cœteris paribus*, for believing the tumor to be fibrous. It may occur in scirrhus of the uterus also. This sound is very distinct from *impulse*. I have seen and felt impulse in ovarian dropsy; but then the sound is not heard. This is especially true in regard to the *double* cardiac sound. Ovarian tumors, except in very exceptional cases, and only where they are solid, if at all, do not transmit the cardiac sounds; fluids being generally non-conductors of sound.

I have said that the tubular souffle is often heard loudest within the vagina. Occasionally it may be heard here with a common straight stethoscope; and if this be long, and made of glass, it is very easily applied. The patient is put on her left side; the nates are brought to the edge of the bed; and the thighs are well flexed on the body. If the woman be not too fat, and have a good perineum, and pressure be made on this part by the stethoscope, the instrument impinges the wall of the vagina against the tumor, and the souffle may be heard through it. In stout women, the stethoscope may be safely introduced *per vaginam*, and made to press upon the tumor; and, because made of glass, the rubbing of the clothes upon it is scarcely heard, so that no exposure is needed.

I have also used a curved stethoscope made of glass with advantage. It may then be passed when the patient lies on her back.

As a rule, however, I prefer the instrument I now show you, which I call a *vaginoscope.* It consists of the ordinary double stethoscope, to the distil end of which is attached a wooden speculum; this may be introduced as a speculum; and the ear-pieces being applied to the ear convey any sounds heard in that region. At any rate, in obscure cases, you will find it useful.

I cannot better exemplify the truth of the preceding statements, than by quoting the following remarkable case:—

Miss C., aged forty-two, had pretty good health up to five years ago, since which period she had not felt so strong. The catamenia had always been regular; but until lately rather under time than over, and lasted five or six days. For the last two years she had been more unwell; the period had lasted a week altogether, lingering a little for the succeeding week; recurring every three weeks or twenty-three days. This increase was due, not to clots, but to an unusual quantity of water, which obliged her to use two napkins at the same time. The function began at the age of 17 or 18. Before 22, she had had dysmenorrhœa for one day, not since.

About three years and a-half ago, on her lifting up a heavy weight, she felt something give way in her side as if she had twisted herself there; and she could not raise herself erect for the pain for a few minutes. Three years ago, while rubbing herself for the stomach-ache, she felt a round tumor in the left iliac region. Even before this she had felt some bearing down in front. There was no alteration in the breasts at the time.

The *breasts* presented no areola. There were some small follicles around, whitish; becoming whiter on tightening sideways.

The *tumor* had enlarged, because formerly she could not feel it in the erect position; while now she could. At times it was very painful; always more so before a period. She thought she could connect the enlargement with the increased catamenial flow, and the color was less dark than it was now.

On *external palpation*, there was dulness for four inches above the pubes. There was a projection half-way between the pubes and umbilicus, of the size of an apple, quite pushing forward the abdominal parietes at this point. This was rounded, and the hand could grasp it superiorly. The dulness extended on a level with this quite to the left side. On the right side, about two inches beyond the tumor, the cæcal region was clear, as well as the two lumbar regions. All the dull parts were hard to the feel. The central projection was, however, movable upon the growth below it. It could be moved on the right side, quite near to Poupart's ligament; on the left side, only half an inch. In either case, when so stretched, tight bands might be felt uniting it to the posterior growth.

*Auscultation.*—On the front of the first growth, extending to the right side, there was quite a musical murmur, with a thrill, ceasing on deep inspiration. Over the projection superiorly, there was no sound; just below it, however, and over the pubes, there was distinct *tubular* souffle. There was a less loud sound on the left part of the posterior growth, and *vesicular.*

*Vaginal and Rectal Examination.*—The os was small, high up behind the pubes; the lips were small. The whole pelvic cavity behind the vagina was filled by a large, hard mass, smooth, somewhat lobulated; at the lower part, it was semi-elastic. Cystic sounding proved that the bladder was rather to the right side. The uterine sound penetrated to its normal length; and anteverted uterus, and turned rather to the right side. The finger in the rectum detected the tumor between it and the vagina, in the posterior *cul-de-sac.*

The *Vaginoscope* gave no indication of murmur. An exploratory puncture made at the inferior portion gave exit to about half an ounce of serum. This proved to be so on microsopical examination. The puncture afterwards bled freely; but the bleeding was arrested by the actual cautery.

The diagnosis was, exra-uterine fibrous tumor, with a pedicle attached to the posterior wall of the uterus, inferiorly infiltrated with serum.—*British Medical Journal, May* 28, 1864, *p.* 579.

